# Commentary: Treatment failure and success: a commentary on defining and treating pediatric treatment‐resistant depression – reflections on Dwyer et al. (2020)

**DOI:** 10.1111/jcpp.13207

**Published:** 2020-02-07

**Authors:** Jeffrey R. Strawn, Paul E. Croarkin

**Affiliations:** ^1^ Department of Psychiatry and Behavioral Neuroscience University of Cincinnati, College of Medicine Cincinnati OH USA; ^2^ Division of Child and Adolescent Psychiatry Department of Pediatrics Cincinnati Children's Hospital Medical Center Cincinnati OH USA; ^3^ Department of Psychiatry and Psychology Mayo Clinic Rochester MN USA

**Keywords:** Depression, major depressive disorder, treatment‐resistant depression

## Abstract

Nearly two in five youth with major depressive disorder fail to respond to first‐line interventions. As such, treatment‐resistant depression represents a formidable challenge for clinicians and researchers. In fact, even considering the diagnosis of treatment‐resistant depression in children and adolescents requires (a) defining treatment‐resistant depression and, by extension, treatment failure; (b) defining recovery; (c) understanding its developmental trajectory; and in addition to (d) understanding the evidence for treatment interventions in this population. Accumulating data suggest that treatment‐resistant depression is heterogeneous and that this heterogeneity may inform interventions. Additionally, these data suggest that substantially more nuance is needed in evaluating the ‘adequacy’ of prior treatments whether they are psychotherapeutic or psychopharmacologic. Last, adjunctive interventions that focus on neuromodulation, glutamatergic, GABAergic, and inflammatory pathways remain poorly understood in youth with treatment‐resistant depression despite very significant advances in adults with treatment‐resistant depression.

## Abstract 2

Read the full article at doi: 10.1111/jcpp.13202

With 40% of depressed youth failing to respond to first‐line interventions, treatment‐resistant depression represents a formidable challenge for clinicians and researchers. In fact, even considering the diagnosis of treatment‐resistant depression in children and adolescents compels us to struggle with difficult‐to‐answer questions. How do we define treatment‐resistant depression and, by extension, treatment failure? What is recovery? Does ‘treatment‐resistant depression’ represents an endophenotype (i.e., core residual symptoms)? And, most importantly, how should we treat these youth?

In this issue, Dwyer, Stringaris, Brent, and Bloch (2020) provide an overview of treatment‐resistant depression in children and adolescents and attempt to answer some of these questions. Their thoughtful review of treatment‐resistant depression in children and adolescents summarizes what is known regarding the prevalence, phenomenology, and risk of treatment‐resistant depression in youth while advocating for more systematic approaches to categorizing and treating these patients, proposing new definitions, and laying the foundation for future research. Importantly, this work raises several key questions that warrant additional discussion.

## How are treatment‐resistant depression and treatment failure best defined?

Youth with treatment‐resistant depression comprises a heterogeneous population. These patients include those who could not tolerate standard treatment, those with residual symptoms *despite* treatment, and those who enjoyed brief – but ephemeral – improvement. This heterogeneity underscores the difficulty in defining treatment‐resistant depression. As noted by Dwyer and colleagues (2020), the Treatment of SSRI Resistant Depression in Adolescents (TORDIA) study (Brent et al., 2008) defined treatment resistance as ongoing depressive symptom severity with a total score on the Children’s Depression Rating Scale‐Revised (CDRS‐R) ≥40 despite at least 8 weeks of treatment with an SSRI at a dose of at least 40 mg (or equivalent dose of another SSRI). Like most work in adults, the definition of adequate treatment has been based on ‘dose’ rather than ‘medication exposure’. This is important in that data over the past decade increasingly illustrate that actual SSRI exposure varies by age, sex, and adherence in adolescents (Ramsey, Bishop, & Strawn, 2019). Moreover, for some of the medications that are commonly used in depressed adolescents, the relationship between dose and blood level varies considerably. For example, a CYP2C19 poor metabolizer requires only 50 mg of sertraline to generate the same blood level as a normal metabolizer who is treated with 150 mg daily (Strawn, Poweleit, & Ramsey, 2019). Thus, recent data raise the possibility that clinicians should look beyond dose in establishing the adequacy of a prior medication trial. Similarly, with regard to the adequacy of psychotherapy, multiple psychotherapies have evidence in adolescents with depressive disorders, including interpersonal, cognitive behavioral, and psychodynamic. Current approaches focus categorically on the presence or absence of a prior psychotherapy trial. In clinical practice, we must consider the frequency of the therapy sessions, the therapist–patient–family alliance, the fit of psychotherapeutic modality to the patient, target symptoms, cognitive flexibility, and attachment style. However, it is important to note that we are not defining the adequacy of the prior psychopharmacologic treatments in terms of whether it reduced symptoms but rather whether the individual factors that maximize its success probability in clinical trials were met. Taken together, accumulating data suggest that substantially more nuance is needed in evaluating the ‘adequacy’ of prior treatments whether they are psychotherapeutic or psychopharmacologic. Moreover, this issue raises the possibility of hierarchies of treatment failures and levels of treatment resistance as Dwyer and colleagues summarized.

In TORDIA, adolescents, who were treated with cognitive behavioral therapy, had ≥2 adequate SSRI trials, or a history of nonresponse to venlafaxine was excluded. While this study provided many clinical insights, larger studies with more participants are important to understand of treatment‐resistant depression and children and adolescents. The definition, phenomenology, ideal diagnostic assessments, and treatment protocols for treatment‐resistant depression are still nebulous. Future work could continue to adapt the antidepressant history treatment form for children and adolescents. This tool is the current standard for interventional trials in treatment‐resistant depression in adults, but has yet to be adapted in pediatric patients to stage treatment‐resistant depression.

## Does ‘treatment‐resistant depression’ represent an endophenotype (i.e., core residual symptoms or specific comorbidity patterns)?

The common view of treatment‐resistant depression – in adults as well as in youth – through a cross‐sectional lens potentially obscures the longitudinal course of treatment‐resistant depression. As Dwyer and colleagues point out, longitudinal symptom assessment is of utmost importance. In fact, the notion of digital phenotyping to quantify moment‐to‐moment fluctuation in affective symptoms, and potentially functioning, may provide ‘objective assessments of symptomatology in the context of patients’ daily lives with continuous measurements’. Emerging work with ecological momentary assessment tools will provide additional tools to further the understanding of treatment‐resistant depression in youth (Forbes et al., 2012). Assessing fluctuations in depressive symptoms over time is not only clinically important but potentially transformative; most studies published in the pages of journals like *Journal of Child Psychology and Psychology,* and others focus on depressive symptoms measured cross‐sectionally by rating scales. By contrast, longitudinal assessments of symptoms might focus on functional outcomes, engagement, and interpersonal aspects of depression and would necessitate tectonic shifts in how researchers, clinicians, and funding agencies interpret these studies and translate findings to the clinic.

Regarding specific endophenotypes, findings from TORDIA and Treatment for Adolescents with Depression Study (TADS) highlight the impact of clinical and demographic heterogeneity on outcomes in adolescent treatment‐resistant depression. For example, socioeconomic status impacts response to psychotherapy (Curry et al., 2006) and trauma, which is ubiquitous in adolescents with treatment‐resistant depression represents another critical factor in precision medicine approaches these youth. Further, specific clinical features dramatically influence outcomes in this population. For example, irritability represents a key feature in treatment‐resistant depression and the impact of chronic irritability on treatment resistance has only recently been explored (Towbin et al., 2020). Contemporary work on the neurobiology and developmental lines of irritability in youth will likely continue to inform on optimal classification and treatment approach for treatment‐resistant depression (Tseng et al., 2019). Taken together, these data raise the possibility that in youth with treatment‐resistant depression, interventions could be tailored based on the prominence of specific symptoms, earlier exposure to adversity, or other factors.

## How should treatment‐resistant depression in youth be treated?

Dwyer and colleagues review the very limited evidence base for treatment‐resistant depression in children and adolescents before moving to interventions that are – at present – not supported by randomized controlled trials in youth. This is not coincidental as the evidence base for treatment‐resistant depression in adolescents consists of fewer than a half dozen randomized, prospective trials. Ongoing interventional work with ketamine and transcranial magnetic stimulation (TMS) both in research settings and clinical practice further underscores the importance of future work. In most areas of the United States, clinical practice with ketamine and TMS is outpacing the evidence base. In current practice at academic medical centers, it is not uncommon to consult on children and adolescents who have received these interventions for depression in the absence of firm history of ‘resistance’ to standard treatments. There are many considerations beyond the unknowns related to the safety and efficacy of these interventions during neurodevelopment. For example, there may be ‘treatment responsive’ ketamine or TMS phenotypes that have not yet been identified. The optimal sequence or algorithm for these interventions is unknown, and current off‐label practices appear to simply follow patient preferences. Further, adjunctive pharmacologic interventions that focus on glutamatergic, GABAergic, and inflammatory pathways remain poorly understood in youth with treatment‐resistant depression despite very significant advances in adults with treatment‐resistant depression.

Dr. Dwyer and colleagues present a lucid review and framework for staging treatment‐resistant depression in adolescents in addition to reviewing potential treatment strategies. Their review underscores the need for precision medicine approaches given the heterogeneity of treatment response in this population as well as the potential endophenotypes within treatment‐resistant depression populations. For clinicians, this translates into important and familiar questions: When considering two youth with treatment‐resistant depression, should a younger patient with co‐occurring anxiety receive a different ‘next step’ intervention compared to an older adolescent with high irritability and a co‐occurring substance use disorder? This review also highlights the problematic nature of defining the adequacy of prior treatments in terms of dose and time instead of accounting for significant differences in medication exposure or the trajectory of response. Further, this review raises questions regarding the clinical validity of the staging approach for treatment‐resistant depression and how such strategies can truly be implemented in the clinic. However, given the dearth of guidelines and data related to treatment‐resistant depression in children and adolescents, this review represents a bold and important step forward.
